# Clinical Impact of the Immunome in Lymphoid Malignancies: The Role of Myeloid-Derived Suppressor Cells

**DOI:** 10.3389/fonc.2015.00104

**Published:** 2015-05-21

**Authors:** Calogero Vetro, Alessandra Romano, Flavia Ancora, Francesco Coppolino, Maria V. Brundo, Salvatore A. Raccuia, Fabrizio Puglisi, Daniele Tibullo, Piera La Cava, Cesarina Giallongo, Nunziatina L. Parrinello

**Affiliations:** ^1^Division of Haematology, AOU “Policlinico – Vittorio Emanuele”, University of Catania, Catania, Italy; ^2^Department of Radiology, University of Palermo, Palermo, Italy; ^3^Department of Biological, Geological and Environmental Sciences, University of Catania, Catania, Italy; ^4^National Research Council Institute for Agricultural and Forest Systems in the Mediterranean, National Research Council, Catania, Italy

**Keywords:** microenvironment, lymphoma, MDSCs, prognostication

## Abstract

The better definition of the mutual sustainment between neoplastic cells and immune system has been translated from the bench to the bedside acquiring value as prognostic factor. Additionally, it represents a promising tool for improving therapeutic strategies. In this context, myeloid-derived suppressor cells (MDSCs) have gained a central role in tumor developing with consequent therapeutic implications. In this review, we will focus on the biological and clinical impact of the study of MDSCs in the settings of lymphoid malignancies.

## Introduction

The study of microenvironment in hematological malignancies is becoming more and more intriguing. New soluble factors and cell-to-cell interactions are under investigation. In this complex network, myeloid-derived suppressor cells (MDSCs) represent a central hub. MDSCs are cells with suppressive habit, able to silence cytotoxic immune-response. This is due to the expression of two key enzymes, Arginase-1 (Arg-1) and the inducible form of nitric oxide synthase (iNOS). As a result, they are able to suppress T-cell response and to modulate regulatory T-cells (T-reg) functions. It is a real revolution in the field of immunological studies, in particular when applied to onco-hematological diseases.

## Pathogenetic and Clinical Importance of Tumor Microenvironment in Lymphoproliferative Disorders

In order to better understand MDSC role in lymphoproliferative diseases, a brief overview on tumor microenvironment is required.

The tumor has been depicted as a mass of neoplastic cells for several years. However, recent studies have been demonstrating, both in solid and hematological tumors, an intricate cross-talk between neoplastic and inflammatory cells (1–3). Indeed, the immune system is able to create a fertile soil where neoplastic cells proliferate (4–7). It is usually composed by Th2 cytokines (such as IL-4, IL-10), angiogenetic factors (COX2, VEGF), and chemokines (CXCL12) (4, 8–10).

However, this cross-talk is not always clear and univocal. For example, compared to lymphoid hyperplasia, patients suffering from Hodgkin lymphoma (HL) have a greater number of CD4+CD25+ cells, T-regulatory (T-reg) markers (11). Additionally, the greater the T-reg amount, the better the prognosis (10, 12, 13). This would be justified by the fact that T-reg cells would be able to regulate the immune response, thus limiting tumor progression. However, the immune-escape and the ability to suppress T-cell response are required for HL development (6, 10) and Hodgkin–Reed Sternberg cells are themselves able to produce immunosuppressive factors, so that the higher the amount of FOXP3 positive cells (a transcription factor expressed by T-reg), the poorer the prognosis (14), being the mirror of the immune-escape of Hodgkin–Reed Sternberg cells. Quite similarly, in multiple myeloma (MM), clinical series indicated that the greater the bone marrow (BM) Treg amount, the more adverse the prognosis (15). However, there are no clear evidences, since in some studies the percentage of Treg into the BM is inferior compared to normal subjects (16), while in other studies, the number is increased (17). Additionally, in other reports, the amount of Treg is greater in the BM than peripheral blood (PB) (18). Indeed, a standard definition of MM features and Treg phenotype would be of great help in function definition (19) and such a perspective study is warranted. It has recently highlighted that the only positivity for FOXP3 expression is not a definitive and unique marker for Treg, being also characterized as CD3^+^CD4^+^CD25^+^CD127^low^ cells (18, 19).

Recently, a new subset of regulatory T-cells have been identified, i.e., Th17. The hallmark of these cells is IL-17 and IL-22 production and they are strictly dependent on IL-21, IL-22, IL-23, IL-27, and IL-6 (16, 20). Th17 amount is augmented in both PB and BM of MM patients. Additionally, MM-plasma cells (PC) express on cell surface the IL-17R (receptor of IL-17) (16) and its amount relate with lytic lesions (21), tumor stage, serum lactate dehydrogenase concentration, and serum creatinine concentration (22). Additionally, this effect is reverted when a Th1 BM enrichment is induced (21).

Apart from the lymphoid axis, an intricate cross-talk exists with the myeloid subset, especially with tumor-associated macrophages (TAMs). TAMs have a pivotal role in regulating lymphoma behavior in several histotypes, including Hodgkin’s lymphoma (23), non-Hodgkin lymphoma (NHL), and follicular lymphoma (FL) (24). In several hematological tumors, macrophages are able to address the behavior of the entire microenvironment and also of neoplastic cells, especially in early stages of the disease (10). This fact makes these cells as essential for tumor promotion phase. By secreting TGF-β and IL-10 (25), suppressing T-cell activation (26), and promoting angiogenesis (25, 27), TAMs favor the immune-escape of the tumor (25, 28, 29). Many studies correlated the increase of TAMs in tumor context with tumor angiogenesis, metastasis, and tumor progression (10, 24, 28–30). Additionally, the amount of TAMs positively relate with the tumor mass and stage.

Thus, it seems that the microenvironment is essential in the early phases of disease development, and continues to guarantee a permissible milieu to neoplastic cells during tumor growth. Thereafter, neoplastic cells become independent from the microenvironment.

Despite initial encouraging observations reported above, lack of reproducibility, discordant and not confirmative recent studies, and inconsistency of scoring are currently considered potential pitfalls for the routine use of TAMs as biomarkers, leading some authors to not agree with the prognostic power of CD68+ infiltrate (31).

## Myeloid-Derived Suppressor Cells and Their Role in Tumor Microenvironment

Myeloid-derived suppressor cells have been recently identified in solid (32) and hematological (33) cancers as a heterogeneous population of immature and mature cells of myeloid origin that are home to the tumors and contribute indirectly to angiogenesis, growth, and metastasis (34). They originate on the BM, but acquire the ability to home into secondary lymphoid organs (35) and also in tumors mass (36, 37). Indeed, they are able to migrate into liver, inducing a suppressive habit to Kupfer cells (through the over-expression of PDL1) able to silence T-cell function through Arginase-1 (Arg-1) release. Furthermore, they are able to migrate into tumor context where they exhibit the immunosuppressive function (35). Interestingly, Arginase activity into tumor context is greater than in PB (35).

This homing capability has two important consequences. First, marking MDSCs; it is possible to follow the homing phase without sacrificing mouse models. Second, MDSCs can be used as therapeutic vehicle (38).

Morphologically, two subsets can be identified: granulocytes (N-MDSCs) or monocytes (mo-MDSCs) (39, 40), possessing, respectively, a polymorphonuclear or monocytic feature. First identification of MDSCs markers was from mouse models where granulocytic MDSCs are identified as CD11b^+^ LY6G^+^ LY6C^low^ cells, while monocytic MDSCs are CD11b^+^LY6G^-^LY6C^high^ (39, 41). Due to the lack of LY6G and LY6C on human cells, the definition of MDSCs in human is still argued. Basically, mo-MDSCs are defined as CD14^+^HLA-DR^low/−^ and G-MDSCs as CD11b^+^CD33^+^CD14^−^HLA-DR^low/−^ (CD15 and/or CD66b can also be positive) (32, 40). Prior studies introduced also a subset of immature MDSCs CD34^+^ (im-MDSCs) (42).

Apart from morphological and immunophenotipic identification, the definition of MDSCs relies on their functional properties and suppressive activities. MDSCs can induce T-cell tolerance through the expression of Arg-1. In fact, starving lymphocytes, depleting Arginine, reduce significantly their function leading to a cell cycle arrest in G0/G1 stage, due to a repression of cyclin production. Contrarily, treatment with Nor-NOHA or NOHA (an Arg-I inhibitor) is able to revert the immune-suppressive ability of MDSCs. Interestingly, this pathway is more pronounced in G-MDSCs (43), while Mo-MDSCs deplete the available arginine through NOS2 activity (39). The overproduction of nitric oxide leads to augmented levels of peroxynitrite and nitrotyrosine. This is peculiar to silence TCR pathways and thus T-cell function (32, 39, 44), promoting the escape from immune-surveillance (34, 45). T and NK cell dysfunction induced by MDSCs can in turn favor the aberrant MDSCs dismissing in a pathological loop (32, 34, 41, 45). Lately, recent investigations suggested that MDSCs are also the progenitors of TAMs in the BM (32, 46). MDSCs can also produce a large amount of cytokines, able to drive the microenvironment toward a pro-tumoral background ambient (35, 46). Producing IL-10, MDSCs can decrease the amount of IL-6 and TNF-α, increasing NO.

The elaborate cross-talk between macrophages, MDSCs, and tumor cells result in differential production of IL-6, IL-10, TNF-α, and NO, suggesting that the interaction between these cells has the potential to significantly alter the inflammatory milieu within the tumor microenvironment (2, 3, 40, 47). This fact makes MDSCs noteworthy to be studied in hematological tumor. Additionally, they would represent a valid and reliable marker to be used in these malignancies since their presence in PB is guaranteed by the homing ability.

## Clinical Progress in MDSC Study and Applications

Studies on MDSCs in hematological malignancies are increasing since the better definition of this cellular subtype and its functional analysis. The peculiar MDSCs function and activity, together with the ability to isolate them from BM and PB has led many groups to focus on their study (48). Translating these evidences to the clinical practice, several studies are emerging with important prognostic and therapeutic consequences in both HL and NHL (2, 3).

Pivotal studies by Lin et al. (49) found that a subpopulation of CD14^+^/HLA-DR^−/low^ monocytes exert immunosuppressive function in NHL patients by depleting arginine bioavailability through Arg-1 activity. In particular, monocytes were able to abrogate Th1 lymphocyte activation to antigen stimulation, with a fivefold decrease in the secretion of IFN-γ. Interestingly, the proliferation rate of patients’ lymphocytes was restored when suppressive monocytes were depleted from the medium. The monocytes were also impaired itself in their functions, due to the reduced levels of pSTAT1 expression and reduced ability to produce IFN-γ. Additionally, the higher the amount of suppressive monocytes, the more adverse the prognosis. The monocytes were also rich in Arg-1 production, and serum from NHL patients had higher levels of ARG-1 compared to control. Additionally, the levels of Arg-1 related with suppressive monocytes. Later, this highlights the identification of MDSCs as central in tumor escape. Indeed, in the A20 murine model, Serafini et al. found that the immune-escape depends strictly on MDSCs [defined as activity that is able to expand selectively Treg (CD25+/FOXP3+)] (50). Interestingly, treatment with l-NMMA and/or NOHA reverted the immunosuppressive feature by suppressing Arg-1 and/or iNOS activity. The combination with both drugs completely reverted the immunosuppressive feature.

Among lymphoma subtypes, diffuse large B-cell lymphoma (DLBCL) has the greatest amount of suppressive monocytes followed by FL and indolent lymphomas (49). Recently, in a limited cohort of 23 DLBCL patients, PB mo-MDSCs have been evaluated as CD14^+^/HLA-DR^−^ cells (51). Additionally, DLBCL patients have a greater amount of Mo-MDSCs on PB compared to healthy control and, when complete remission is achieved, their values return to be equal to healthy control. However, the small number of patients did not lead the authors to conclude a direct correlation between monocytosis and mo-MDSCs. Interestingly, only three patients among a total of five patients with monocytosis showed augmented levels of Mo-MDSCs.

In HL, the inflammation is the hallmark of the disease (52). HL patients have a greater amount of MDSCs compared to healthy control (53–55) and, to date, we have been able to identify the three main MDSCs subsets, i.e., G-MDSCs, Mo-MDSCs, and im-MDSCs. However, only CD34+ cells (im-MDSCs) seem to relate with prognosis in HL patients, even though treated up-front with a risk-adapted therapy (56). At diagnosis, HL patients showed higher levels of MDSCs subsets compared to matched healthy controls. Additionally, the greater the tumor mass, the greater the MDSCs count, reflecting the disease stage and aggressiveness. Regarding the clinical outcome, after 34 months of follow-up, im-MDSCs were the only subset related to clinical outcome. On the contrary, mo-MDSCs and G-MDSCs failed to show a prognostic impact. In particular, setting a cut-off level of 4.5 cells/μL, im-MDSCs showed a sensitivity and specificity >85% and a specificity greater than 70% in both early and advanced stage disease. Median progression-free survival (PFS) in patients with high levels of MDSCs was 14.7 months compared to a not reached median of the low count group. The prognostic value of im-MDSCs was retained also applying the multivariate analysis. In addition, MDSCs count can add information to the most important prognostic factor in HD, i.e., positive positron emission tomography (PET) after two cycles of chemotherapy (PET-2). An additional advantage of im-MDSCs count over PET-2 would be the availability at diagnosis. Moreover, the combination of MDSCs count and PET-2 evaluation allows to define three different groups of patients with different outcome (56).

## Conclusion

The study of the immunome has become more and more intricate with new factors and cell-to-cell interactions discovered. An emerging role is played by the study of the myeloid dysfunction as a central hub in the complex network depicting the disease. Even if more studied on solid tumors, MDSCs are a reliable tool also in hematological malignancies, in particular in the setting of lymphoid malignancies. Additionally, the emerging reports in clinical settings are making them worth to be studied and used as prognostic factor.

## Conflict of Interest Statement

The authors declare that the research was conducted in the absence of any commercial or financial relationships that could be construed as a potential conflict of interest.
